# Role of Glucose in IRS Signaling in Rat Pancreatic Islets: Specific Effects and Interplay with Insulin

**DOI:** 10.1080/15438600490905169

**Published:** 2004

**Authors:** Maryline Paris, Catherine Bernard-Kargar, José Vilar, Nadim Kassis, Alain Ktorza

**Affiliations:** 1 Laboratoire de Physiopathologie de la Nutrition Université Paris 7 Paris France; 2 Physiologie et Endocrinologie Cellulaire et Moléculaire Rénale INSERM U356 Paris France; 3 IDRS 11, rue des Moulineaux Suresnes 92150 France

## Abstract

We investigated the possible interplay between insulin and glucose signaling pathways in rat pancreatic *β*-cell with a special focus on the role of glucose in IRS signaling *in vivo*. Three groups of rats were constituted by combining simultaneous infusion during 48 h either of glucose and/or insulin, or glucose+diazoxide: Hyperglycemic- Hyperinsulinemic (HGHI), euglycemic-Hyperinsulinemic (eGHI), Hyperglycemic-euinsulinemic (HGeI). Control rats were infused with 0,9% NaCl. In HGHI and HGeI rats plasma glucose levels were maintained at 20-22 mmol/l. In eGHI rats, plasma glucose was not different from that of controls, whereas plasma insulin was much higher than in controls. In HGHI rats, IRS-2 mRNA expression, total protein and phosphorylated protein amounts were increased compared to controls. In HGeI rats, only IRS-2 mRNA expression was increased. No change was observed in eGHI rats whatever the parameter considered. In all groups, mRNA concentration of IRS-1 was similar to that of controls. The quantity of total and phosphorylated IRS- 1 protein was dramatically increased in HGHI rats and to a lesser extent in eGHI rats. Neither mRNA nor IRS-1 protein expression were modified in HGeI rats. The data suggest that glucose and insulin play at once a specific and a complementary role in islet IRSs signaling. Especially, glucose stimulates IRS-2 mRNA expression whatever the insulin status and independently of the secretory process. The differential regulation of IRS-1 and IRS-2 expressions is in agreement with their supposed different involvement in the control of *β*-cell growth and function.


*Experimental Diab. Res.*, 5:257–263, 2004
Copyright © Taylor and Francis Inc.
ISSN: 1543-8600 print / 1543-8619 online
DOI: 10.1080/15438600490905169

Taylor & Francis
Taylor & Francis Group

### Role of Glucose in IRS Signaling in Rat Pancreatic Islets: Specific Effects and Interplay with Insulin

**Maryline Paris,[1] Catherine Bernard-Kargar,[1] José Vilar,[2] Nadim Kassis,[1] and Alain Ktorza[1,3]**

[1]*Laboratoire de Physiopathologie de la Nutrition, Université Paris 7, Paris-France*
[2]*Physiologie et Endocrinologie Cellulaire et Moléculaire Rénale, INSERM U356 Paris-France*
[3]*IDRS, Suresnes, France*

**We investigated the possible interplay between insulin and glucose signaling pathways in rat pancreatic $\beta$-cell with a special focus on the role of glucose in IRS signaling *in vivo*. Three groups of rats were constituted by combining simultaneous infusion during 48 h either of glucose and/or insulin, or glucose+diazoxide: Hyperglycemic-Hyperinsulinemic (HGHI), euglycemic-Hyperinsulinemic (eGHI), Hyperglycemic-euinsulinemic (HGeI). Control rats were infused with 0,9% NaCl. In HGHI and HGeI rats plasma glucose levels were maintained at 20-22 mmol/l. In eGHI rats, plasma glucose was not different from that of controls, whereas plasma insulin was much higher than in controls. In HGHI rats, IRS-2 mRNA expression, total protein and phosphorylated protein amounts were increased compared to controls. In HGeI rats, only IRS-2 mRNA expression was increased. No change was observed in eGHI rats whatever the parameter considered. In all groups, mRNA concentration of IRS-1 was similar to that of controls. The quantity of total and phosphorylated IRS-1 protein was dramatically increased in HGHI rats and to a lesser extent in eGHI rats. Neither mRNA nor IRS-1 protein expression were modified in HGeI rats. The data suggest that glucose and insulin play at once a specific and a complementary role in islet IRSs signaling. Especially, glucose stimulates IRS-2 mRNA expression whatever the insulin status and independently of the secretory process. The differential regulation of IRS-1 and IRS-2 expressions is in agreement with their supposed different involvement in the control of $\beta$-cell growth and function.**

**Keywords** Glucose; Insulin; IRSs Signaling; Pancreatic Islets; *In vivo*

### INTRODUCTION

According to our current knowledge, signals generated by glucose metabolism within pancreatic $\beta$-cells are crucial not only for insulin synthesis, storage and release [1], but also for the control of $\beta$-cell growth and $\beta$-cell development [2, 3]. More recently, this concept has been enriched by the discovery of new pathways involved in the maintenance of a functional $\beta$-cell mass. Indeed, the importance of the insulin/insulin-like growth factor (IGF) signaling system for endocrine pancreas function and $\beta$-cell mass homeostasis emerged from several studies using either transgenic mice [4] or $\beta$-cell lines [5].

A possible cross talk between both signaling systems is suggested by recent data showing that metabolic and ionic events, especially calcium-regulated events, currently associated with glucose-induced insulin secretion could also be due to activation of the insulin/IGF signaling system. For example, over expression of the insulin receptor or the insulin-receptor substrate-1 (IRS-1) in $\beta$-cells leads to a high increase in basal cytosolic $Ca^{2+}$ levels resulting from inhibition of $Ca^{2+}$ uptake by endoplasmic reticulum [6]. Moreover, the elevation of cytosolic $Ca^{2+}$ concentration linked to insulin stimulatory process is blunted in $\beta$-cell lines derived from IRS-$1^{-}/^{-}$ mice [7]. Although some studies suggest that glucose may stimulate IRS protein phosphorylation *in vitro* [8, 9], the ability of glucose

Address correspondence to: Alain Ktorza IDRS, 11, rue des Moulineaux. 92150-Suresnes, France. E-mail: alain.ktorza@fr.netgrs.com

*per se* to interfere with insulin/IGF signaling system within pancreatic islets is less well documented and remains to be clarified.

In our study we attempted to evaluate the specific effect of glucose and the combined effects of glucose and insulin on insulin/IGF signaling system in rat $\beta$-cells. We concentrated on IRS-1 and IRS-2 molecules because they are key mediators in insulin and IGF-1 signaling and their importance in $\beta$-cell physiology is evidenced by several recent data [10–12]. Finally, to assess physiological relevance of the data we aimed at performing the experiments *in vivo*. In this way we designed an experimental model in rats consisting in the infusion during 48 h of either glucose to induce hyperglycemia and hyperinsulinemia, or glucose+insulin to provoke hyperinsulinemia associated with euglycemia, or glucose+diazoxide (a potent inhibitor of insulin secretion) to establish hyperglycemia with euinsulinemia. Islet IRS-1 and IRS-2 mRNA, protein expression and phosphorylation were further evaluated by semi-quantitative RT-PCR, western blotting and immunoprecipitation, respectively.

### RESEARCH DESIGN AND METHODS

### Animals

Three-month-old male Wistar rats weighing 280-300g, were used. They had free access to water and standard laboratory diet pellet (No.113, UAR, Villemoisson-sur-Orge, France).

### Infusions

Rats were randomly divided into five groups as follows: 1. 0.9% NaCl infused rats (controls; NaCl rats), 2. Glucose-infused rats (hyperglycemic-hyperinsulinemic; HGHI rats), 3. Glucose+insulin-infused rats (euglycemic-hyperinsulinemic; eGHI rats), 4. diazoxide-infused rats (control diazoxide; DZ rats), 5. glucose+diazoxide-infused rats (hyperglycemic-euinsulinemic; HGeI rats). The long-term infusion technique in unrestrained rats was used, as previously described [13, 14]. Briefly, 2 days before infusion rats were fitted with an indwelling jugular vein catheter, and during the infusion period, rats were permanently connected to a pump via a device fitted with a water-tight swivel. All infusions lasted 48 h.

In HGHI rats, hypertonic (30% wt/vol) glucose (Chaix & Du Marais, Paris, France) was infused at an initial rate of 50 $\mu$l/min to produce hyperglycemia around 20 mM throughout the infusion period. In eGHI group, euglycemia and hyperinsulinemia were induced using insulin infusion during 48 h at a rate of 30 $\mu$U/min (Novo/Nordisk, Bagsvaerd, Denmark), to produce a hyperinsulinemia ~15 fold higher than that of HGHI rats. Simultaneous glucose infusion allowed to maintain euglycemia (~5 mM). In HGeI rats, hyperglycemia and euinsulinemia were induced by a simultaneous infusion of diazoxide (Sigma, St Louis, USA) and hypertonic (30% wt/vol) glucose, which was infused at the same flow rate as in HGHI rats. Diazoxide solution (added to a bicarbonate-phosphate buffer pH 9.5) was infused in HGeI and the DZ groups at a flow rate of 5 $mg\cdot kg^{-1}\cdot h^{-1}$.

During infusion periods, plasma glucose and insulin concentrations were measured on arterio- venous blood collected from tail vessels by tail snipping, five times daily in HGHI, HGeI, and eGHI rats. This daily control allowed glycemia and insulinemia to be maintained in the required ranges by adjusting the infusion flow rate. Glycemia and insulinemia remained stable in NaCl and DZ groups, so blood was collected only twice daily. Rats in which glycemia and insulinemia did not stay within wished ranges were discarded.

### Islet Isolation

Rats were anesthetized with pentobarbital (4 mg/100g body wt i.p.). Islets of Langerhans were isolated after collagenase digestion of the pancreas according to the method of Pralong et al [15].

### REVERSE TRANSCRIPTION-POLYMERASE CHAIN REACTION (RT-PCR) ASSAY

Total cellular RNA from frozen islets were extracted using Chomczynski's method [16]. 1200 islets were homogenised in trizol reagent (Gibco BRL, Life Technologies Inc., Gaithersburg, USA) with a syringe (700 islets/ml) according to the manufacturer's protocol. RNA were resuspended in distilled water and stored at −80°C until used.

For the semi-quantitative analysis for mRNA encoding IRS-1, IRS-2 and $\alpha$-tubulin (co-amplified to normalize), 1 $\mu$g of total RNA was reversed transcribed and amplified according to the protocol of One Step PCR (Qiagen, Courtaboeuf, France). The primers used for IRS-2 were: forward 5′ GTC-GTT-GTC-TCC-ACC-ACC-3′ and reverse 5′-GTT-CCT-CAG-CCT-TCC-TCT-3′ and provided amplification of a 696pb fragment. The primers used for IRS-1 were: forward 5′-ACC-ATG-GGG-ACA-AGC-CCG-GCC-3′ and reverse 5′-GGG-GCT-GCT-GGT-GTT-GGA-ATC-3′ and provided amplification of a 744 pb fragment. The primers used for the $\alpha$-tubulin were: forward 5′-ATG-CCC-TCA-CCC-ACG-TAC-3′ and reverse 5′-CTC-GCA-TCC-ACT-TCC-CTC-3′ and provide amplification of a 451 pb fragment. cDNA were analyzed after 32 cycles of PCR, allowing an amplification of products within the linear range of the PCR. Each amplification cycle consisted of 1 min denaturation at 94°C, 1 min annealing at 55°C and 1 min extension at 72°C. PCR products were submitted to electrophoresis on a 2% agarose gel and bands were visualized by ethidium bromide staining. Polaroid 665 photographs of the gel were scanned and band intensities were quantitated by densitometry using NIH1.62 Image software (version 1.62; National Institutes of Health, Bethesda, Maryland, USA). Results were expressed as the ratio of IRS-2 or IRS-1 value against $\beta$-tubulin value.

### Immunoprecipitation

Frozen islets were lysed for 5 min at 4°C, in 500$\mu$l lysis buffer (50mM Tris-HCl pH 7.4; 100mM NaCl; 1% nonidet P-40; 5 mM EDTA; 50 mM NaF; 1mM sodium orthovanadate), in the presence of a protease inhibitor cocktail (Roche-diagnostic, Mannheim, Germany). Insoluble material was removed by centrifugation at 12.000 g at 4°C for 5 min. Protein concentration in the supernatant was determined by Lowry's method using commercial kit (Interchim, Montluçon, France). Islet protein (250 $\mu$g) was incubated overnight at 4°C with 2 $\mu$g of anti-IRS-2 antibody (Upstate Biotechnology, Mundolsheim, Germany). Immune complexes were captured by adding protein A-Agarose beads (Upstate Biotechnology, Mundolsheim, Germany) and incubating for 2 h at 4°C. After 1 min centrifugation at 12.000 g at 4°C, the supernatant was removed and stored at −80°C or immediatly immunoprecipitated with 2 $\mu$g IRS-1 antibody (Upstate Biotechnology, Mundolsheim, Germany). Immune complexes in the pellet were briefly washed six times at 12.000g at 4°C with lysis buffer. The pellet was finally suspended in 30 $\mu$l of Laemmeli sample buffer X4 (62 mM Tris-HCl pH6.8, 10% glycerol, 2% SDS, 0.25% bromophenol blue and 1% $\beta$-mercaptoethanol), and boiled for 5 min.

For Western blot analysis, proteins were run on a 10% SDS-Page gel and they were then transferred to nitrocellulose membranes (Amersham, Les Ulis, France). IRS-1 and IRS-2 phosphorylation was analyzed by subsequent preincubation for 30 min in blocking solution (0.2% Tween-20 in PBS) and incubated with 2 $\mu$g of anti-phosphotyrosine antibody (Upstate Biotechnology, Mundolsheim, Germany). The membranes were then washed in PBS containing 0.05% Tween-20 and reincubated with a 5 $\mu$g of horseradish peroxidase-coupled rabbit anti-mouse immunoglobulin G (Upstate Biotechnology, Mundolsheim, Germany). Immunoreactivities were revealed using the ECL chemi-luminescence reaction (Amersham Parmacia Biotech, France). The membranes were then stripped 30 min at 65°C in stripping buffer (Urea 10mM, Sodium phosphate 0.05 M pH6.5, $\beta$-mercaptoethanol 0.1 M). After several washes the membrane were blocked 30 min in PBS with 3% non-fat milk and immunoblotted with 2 $\mu$g anti-IRS-2 antibody or 2 $\mu$g anti-IRS1 antibody (Upstate Biotechnology). Bound antibodies were detected with 5 $\mu$g horseradishh peroxidase-coupled goat anti-rabbit immunoglobulin G (Chemicon International, USA), using the ECL detection system.

The absence of IRS-1 band on membranes obtained after IRS-2 immunoprecipitation and the absence of IRS-2 band on membrane obtained after IRS-1 immunoprecipitation were used as controls for immunoprecipitation, respectively. Band intensities were quantified with NIH1.6 Image.

### Analytical Methods

Plasma glucose was determined by a Glucose Analyzer (Glucotrend, Boehinger Manheim, Germany). Insulin was measured by a radioimmunoassay kit (Sorin, Italy). The lower limit of the assay was 0.07 nmol/l, with a coefficient of variation within and between assay of 6%.

### Data Presentation and Statistical Methods

Data are presented as means ±SE. Statistical significance was determined with the analysis of variance test (ANOVA test). $P < 0.05$ was considered significant.

### RESULTS

### Plasma Glucose and Insulin Concentrations During Infusion

In the NaCl group, plasma glucose and plasma insulin remained stable during the 48 h infusion period (Figure 1). In HGHI rats, glucose infusion led to a rapid increase in glycemia, which was maximal at 6 h and stabilized at 20-22 mmol/l until the end of infusion. As a result, insulinemia increased rapidly reaching a mean value around 1 nmol/l (Figure 1). In the eGHI group, plasma insulin level was 15 times higher than in HGHI rats, whereas plasma glucose was similar to that of NaCl rats (Figure 1). In HGeI rats, insulinemia was maintained close to basal values throughout glucose infusion. The plasma glucose level was as expected to be very similar to that of HGHI group.

Diazoxide infusion did not influence plasma parameters since no differences in both glycemia or insulinemia were observed in the diazoxide group as compared with NaCl rats (Figure 1).

### Pancreatic Islet IRS-2 and IRS-1 mRNA Levels

In HGHI and HGeI groups an increase in the mRNA IRS-2 level was observed (+44% and +63%, respectively) as compared with NaCl group. No modification was observed in eGHI and DZ groups (Figure 2A).

There was no significant modification of the mRNA IRS-1 level in any groups as compared with NaCl rats (Figure 2B).

### IRS-2 and IRS-1 Protein and Phosphorylated Protein Expressions

A 3-fold increase of IRS-2 protein expression was observed in the HGHI group as compared with NaCl group, whereas in the

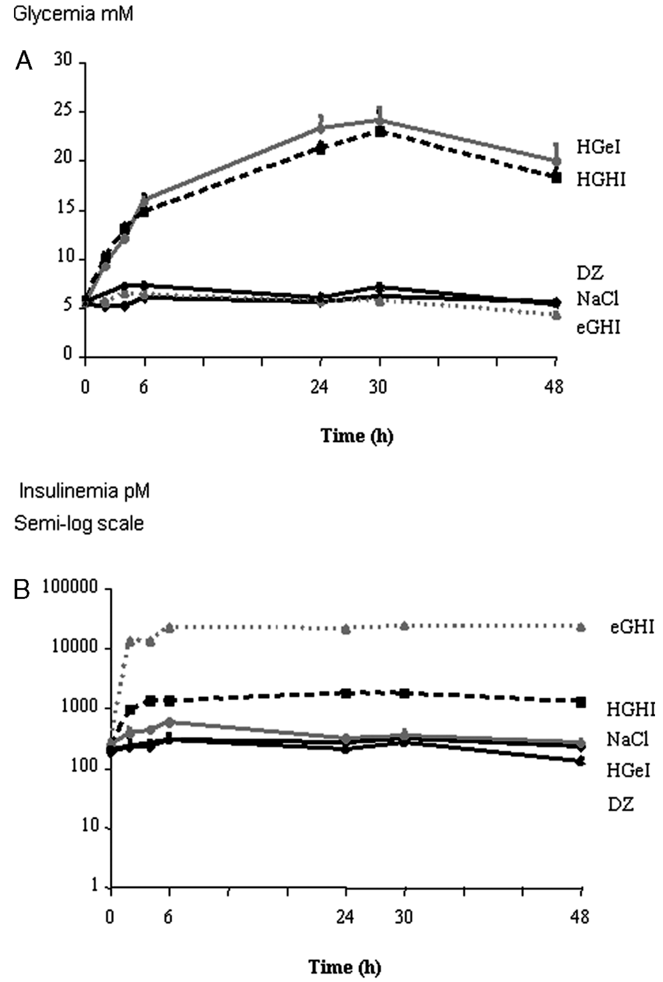

**FIGURE 1**
Time course of plasma glucose and insulin concentrations during a 48 h infusion either saline (NaCl), or glucose (HGHI), or glucose+Insuline (eGHI) or glucose +diazoxide (HGeI). Data are means ± SE. n = 8 in each group. *p < 0.01% compared with saline control.

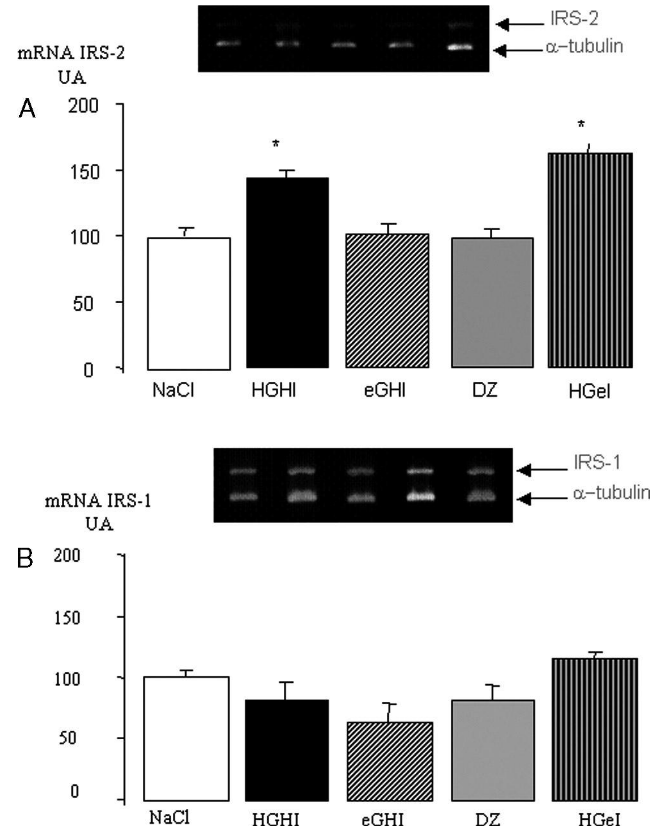

**FIGURE 2**
Expression of IRS-2 (2A) and IRS-1 (2B) mRNA in pancreatic islets after a 48 h infusion either of saline (NaCl), or glucose (HGHI), or glucose + insulin (eGHI), or diazoxide (DZ), and glucose + diazoxide (HGeI). (2A) n = 4 for NaCl , DZ and HGeI, n = 6 for HGHI, n = 4 for eGHI. Data are means ± SE. *P < 0,05; (2B) n = 7 for NaCl, n = 4 for HGHI,eGHI and HGeI; n = 5 for DZ.Data are means ± SE. *P < 0,05

other groups IRS-2 protein expression level remained similar to that of controls (Figure 3A). The phosphorylated IRS-2 level was 1.5-fold higher in HGHI than in NaCl rats. No variation in this parameter was observed in the other groups compared to control NaCl rats (Figure 4A).

As compared with NaCl rats, a 2.5 and 1.5-fold increase in IRS-1 protein expression was noticed in the HGHI and eGHI group respectively. No modification was observed in HGeI group (Figure 3B). Compared with NaCl rats, the IRS-1 activation profile was fitted to the one of IRS-1 protein expression i.e., 2- and 1.5-fold increase in the HGHI and eGHI groups, respectively (Figure 4B). Note that for all parameters of IRS mRNA and protein expression no difference could be detected between DZ and NaCl control rats.

### DISCUSSION

Our data clearly show that islet mRNA and protein IRS expression are influenced by changes in plasma glucose and insulin concentrations *in vivo*. However, both molecules played either specific or synergistic roles according to the IRS isoform considered and the level of control of its expression.

In our opinion, the most striking finding emerging from our study is the demonstration of a specific effect of glucose on islet IRS-2 mRNA expression. At first, islet IRS-2 mRNA concentration was significantly increased in both hyperglycemic-hyperinsulinemic and hyperglycemic-euinsulinemic rats, thus stressing that the stimulating effect of glucose could be exerted independently of the insulin status. Some previous studies dealt with the ability of glucose to interfere with islet insulin/IGF signaling system. Velloso et al. [9] showed that glucose could promote phosphorylation of the insulin receptor and IRS-1 and IRS-2 in a dose-dependent manner in cultured rat pancreatic

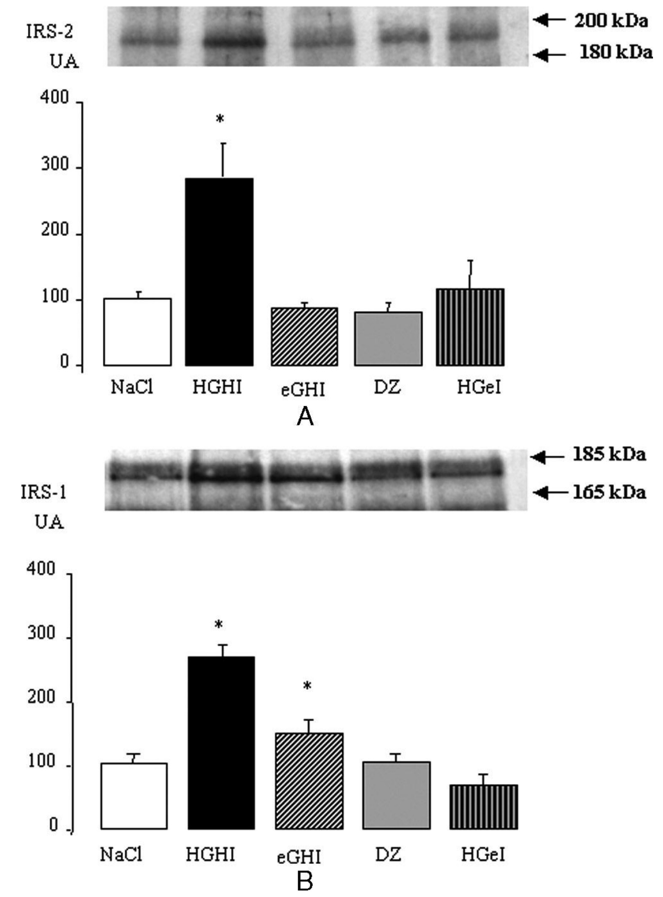

**FIGURE 3**
Protein expression of IRS-2 (3A) and IRS-1 (3B) in pancreatic islets after 48 h infusion of saline (NaCl), glucose (HGHI), glucose+insulin (eGHI), diazoxide (DZ) or glucose +diazoxide (HGeI). (3A) n = 4 for NaCl,HGHI and eGHI; n = 6 DZ; n = 5 for HGeI. Data are means ± SE. *P < 0,05; (3B) n = 7 for NaCl, n = 4 for HGHI, eGHI and HGeI; n = 5 for DZ. Data are means ± SE. *P < 0,05

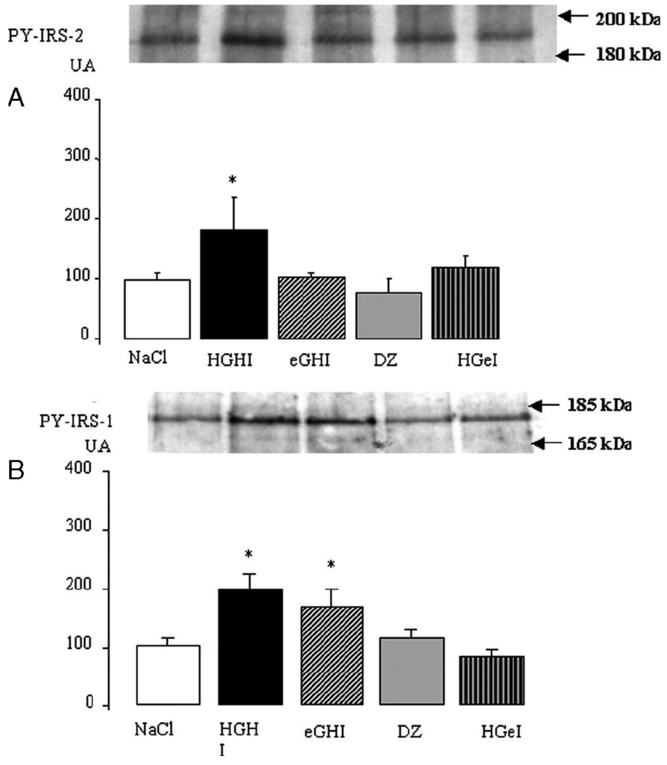

**FIGURE 4**
Expression of tyrosine phosphorylated IRS-2 (4A) and IRS-1 (4B) in pancreatic islets after 48 h infusion of either saline (NaCl), or glucose (HGHI), or glucose+insulin (eGHI), or diazoxide (DZ) or glucose + diazoxide (HGeI). (4A) n = 4 for NaCl, HGHI and eGHI; n = 6 for DZ; n = 5 for HGeI. Data are means ± SE. *P < 0,05; (4B) n = 7 for NaCl, n = 4 for HGHI,eGHI and HGeI; n = 5 for DZ. Data are means ± SE. *P < 0,05

islets. Rothenberg et al. [8] using the $\beta$TC3 insulin secreting $\beta$-cell line demonstrated that glucose-induced insulin secretion promoted phosphorylation of the insulin receptor and its intracellular signal transduction pathway including IRSs molecules. However, in both experiments because high glucose concentration activated the insulin secretory process, glucose-induced insulin secretion rather than glucose itself could be responsible for the activation of the receptor tyrosine kinase signaling by an autocrine mechanism. It is noteworthy that in the Rothenberg et al. [8] study, receptor phosphorylation was prevented by blocking the insulin secretory process but was stimulated by adding insulin to the incubation medium. In our study, the fact that the effect of glucose persisted when insulin secretion was blocked by diazoxide strongly suggests - for the first time to our knowledge - that the increase in islet IRS-2 mRNA expression was not related to the glucose-induced stimulation of insulin secretion but to the stimulating effect of glucose (or glucose metabolism products) itself. Although this provide evidence that glucose and/or glucose metabolism products are involved in the control of the steady-state islet IRS-2 mRNA levels, further investigations are required to determine whether this control is exerted at the level of gene transcription and/or by stabilizing mRNA [17]. Moreover, an indirect effect through the activation of transcription factors by glucose responses cannot be excluded [17].

The association of hyperinsulinemia and hyperglycemia was required for the increase in IRS-2 protein and its phosphorylation. These data are in favor of additional effect of glucose and insulin on IRS-2 protein expression and tyrosine phosphorylation and suggests that insulin in the presence of high glucose could stimulate pancreatic islet IRS-2 gene translation. Whereas the regulation of IRSs proteins activation by phosphorylation is abundantly documented (review in 18), little is known about the control of IRSs proteins expression at least in pancreatic islets. Although direct evidence of the role of insulin did not emerge from these studies, several recent data showed that IRS-2 over expression and/or tyrosine phosphorylation in the pancreas correlates with intense $\beta$-cell growth as well in regenerating pancreas of partially pancreatectomized rats [19] as in pancreatic $\beta$-cell lines [20, 21]. The role of IGF-1 has been explored because of the very likely interface between the insulin and IGF islet signaling pathway (22 and review in 12). In Lingohr et al. study, the increase in IRS-2 expression was specifically related to IGF-1-stimulated $\beta$-cell proliferation.

Moreover the stimulating effect of IGF-1 was glucose-dependent [21]. Withers et al. [22] proposed that IRS-2 was the main mediator of IGF-1 in promoting $\beta$-cell growth during embryogenesis and post-natal development. In our study, we cannot rule out the possibility that insulin effects shown in HGHI and eGHI rats could be ascribed, at least in part, to binding of the hormone to IGF-I binding sites present on pancreatic $\beta$-cells due to the high levels of insulin in euglycemic-hyperinsulinemic rats. We infused insulin at a high flow rate in this group because exogenous infusion of insulin cannot mimic exactly the effect of endogeneous hyperinsulinemia on the $\beta$-cell. Especially the increase in intraislet insulin concentration in response to glucose stimulation with possible autocrine and/or paracrine effects are missed. Previous studies showed that the dramatic increase in intra-islet insulin induced by high glucose was crucial for autocrine and paracrine interactions [23, 24]. Therefore the high flow rate of insulin infusion meets an attempt to increase insulin concentration enough to come near the autocrine/paracrine and endocrine situation induced by glucose infusion in HGHI rats.

The regulation of islet IRS-1 expression by glucose and insulin was quite different from that of IRS-2. Firstly, neither insulin nor glucose influenced islet IRS-1 mRNA levels. Secondly, IRS-1 protein expression and tyrosine phosphorylation were increased by hyperinsulinemia associated or not with hyperglycemia, whereas in hyperglycemic-euinsulinemic rats no change in these parameters was observed. This indicates that in pancreatic islets, IRS-1 protein expression and tyrosine phosphorylation are dependent on insulin levels and not on glucose concentration. In keeping with these data Rothenberg et al. [8] showed that glucose itself was not required for the increase in IRS-1 tyrosine phosphorylation whereas insulin secretion (likely via a paracrine regulation) and/or exogenous insulin were crucial for this increase. To know whether the stimulating effect on IRS-1 expression that we observed strictly reflects insulin action through the binding on its own receptor or is partly due to insulin binding on IGF-1 receptor remains questionable for the same reasons as discussed above for the regulation of IRS-2 expression.

The differential regulation of islet IRS-1 and IRS-2 expression that we observed fits to our current knowledge and hypothesis on the respective biological effects of IRS-1 and IRS-2. There is now a large amount of evidence indicating that IRS-1 and IRS-2 have distinct roles in the maintenance of a functional $\beta$-cell mass, IRS-2 being involved in $\beta$-cell growth and survival [11, 25, 26] whereas IRS-1 signaling is important for the insulin secretory pathway [25, 27, 28].

In conclusion, the data suggest that glucose and insulin play both a specific and a complementary role in islet IRS signalling *in vivo*. Especially, glucose stimulates IRS-2 mRNA expression, whatever the insulin status and independently of the secretory process. The differential regulation of IRS-1 and IRS-2 expressions is in agreement with their supposed different involvement in the control of $\beta$-cell growth and function. Exploring the interplay between glucose and the insulin/IGF signaling system in the $\beta$-cell will help us to better understand the deterioration of $\beta$-cell function and growth in type 2 diabetes. Our model appears as particularly suitable to such studies *in vivo*.

### REFERENCES

[1] Matschinsky, F. M. (1996) Banting Lecture 1995. A lesson in metabolic regulation inspired by the glucokinase glucose sensor paradigm. *Diabetes* **45,** 223–241.

[2] Bonner-Weir, S. (2001) Beta-cell turnover: its assessment and implications. *Diabetes* 50 Suppl **1,** S20–24.

[3] Bernard-Kargar, C., and Ktorza, A. (2001) Endocrine pancreas plasticity under physiological and pathological conditions. *Diabetes* 50 Suppl **1,** S30–35.

[4] Nakae, J., Kido, Y., Kitamura, T.,and Accili, D. (2001) Glucose homeostasis: lessons from knouck-out mice. *Curr.op.endocrinol.diab* **8,** 82–87.

[5] Burks, D. J., and White, M. F. (2001) IRS proteins and beta-cell function. *Diabetes* 50 Suppl **1,** S140–145.

[6] Xu, G. G., Gao, Z. Y., Borge, P. D., and Wolf, B. A. (1999) Insulin receptor substrate 1-induced inhibition of endoplasmic reticulum Ca2+ uptake in beta-cells. Autocrine regulation of intracellular Ca2+ homeostasis and insulin secretion. *J. Biol. Chem.* **274,** 18067–18074.

[7] Aspinwall, C. A., Qian, W. J., Roper, M. G., Kulkarni, R. N., Kahn, C. R., and Kennedy, R. T. (2000) Roles of insulin receptor substrate-1, phosphatidylinositol 3-kinase, and release of intracellular Ca2+ stores in insulin-stimulated insulin secretion in beta -cells. *J. Biol. Chem.* **275,** 22331–22338.

[8] Rothenberg, P. L., Willison, L. D., Simon, J., and Wolf, B. A. (1995) Glucose-induced insulin receptor tyrosine phosphorylation in insulin-secreting beta-cells. *Diabetes* **44,** 802–809.

[9] Velloso, L. A., Carneiro, E. M., Crepaldi, S. C., Boschero, A. C., and Saad, M. J. (1995) Glucose- and insulin-induced phosphorylation of the insulin receptor and its primary substrates IRS-1 and IRS-2 in rat pancreatic islets. *FEBS Lett* **377,** 353–357.

[10] Araki, E., Lipes, M. A., Patti, M. E., Bruning, J. C., Haag, B., Johnson, R. S., et al. (1994) Alternative pathway of insulin signalling in mice with targeted disruption of the IRS-1 gene. *Nature* **372,** 186–190.

[11] Withers, D. J., Gutierrez, J. S., Towery, H., Burks, D. J., Ren, J. M., Previs, S., et al. (1998) Disruption of IRS-2 causes type 2 diabetes in mice. *Nature* **391,** 900–904.

[12] White, M.-F. (2002) IRS proteins and the common path to diabetes. *Am J Physiol. Endocrinol. Metab.* **283,** E413–422.

[13] Ktorza, A., Girard, J. R., Kinebanyan, M. F., and Picon, L. (1981) Hyperglycaemia induced by glucose infusion in the unrestrained pregnant rat during the last three days of gestation: metabolic and hormonal changes in the mother and the fetuses. *Diabetologia* **21,** 569–574.

[14] Bernard, C., Berthault, M. F., Saulnier, C., and Ktorza, A. (1999) Neogenesis vs. apoptosis As main components of pancreatic beta cell mass changes in glucose-infused normal and mildly diabetic adult rats. *Faseb. J.* **13,** 1195–1205.

[15] Pralong, W. F., Bartley, C., and Wollheim, C. B. (1990) Single islet beta-cell stimulation by nutrients: relationship between pyridine nucleotides, cytosolic Ca2+ and secretion. *Embo. J.* **9,** 53–60.

[16] Chomczynski, P., and Sacchi, N. (1987) Single-step method of RNA isolation by acid guanidinium thiocyanate-phenol-chloroform extraction. *Anal. Biochem.* **162,** 156–159.

[17] Vaulont, S., Vasseur-Cognet, M., and Kahn, A. (2000) Glucose regulation of gene transcription. *J. Biol. Chem.* **275,** 31555–31558.

[18] White, M.-F., and Kahn, C. R. (1994) The insulin signaling system. *J. Biol. Chem.* **269,** 1–4.

[19] Jetton, T.-L., Liu, Y. Q., Trotman, W. E., Nevin, P. W., Sun, X. J., and Leahy, J. L. (2001) Enhanced expression of insulin receptor substrate-2 and activation of protein kinase B/Akt in regenerating pancreatic duct epithelium of 60 %-partial pancreatectomy rats. *Diabetologia* **44,** 2056–2065.

[20] Schuppin, G. T., Pons, S., Hugl, S., Aiello, L. P., King, G. L., White, M., et al. (1998) A specific increased expression of insulin receptor substrate 2 in pancreatic beta-cell lines is involved in mediating serum-stimulated beta-cell growth. *Diabetes* **47,** 1074–1085.

[21] Lingohr, M. K., Dickson, L. M., McCuaig, J. F., Hugl, S. R., Twardzik, D. R., and Rhodes, C. J. (2002) Activation of IRS-2-mediated signal transduction by IGF-1, but not TGF-alpha or EGF, augments pancreatic beta-cell proliferation. *Diabetes* **51,** 966–976.

[22] Withers, D. J., Burks, D. J., Towery, H. H., Altamuro, S. L., Flint, C. L., and White, M. F. (1999) Irs-2 coordinates Igf-1 receptor-mediated beta-cell development and peripheral insulin signalling. *Nat. Genet.* **23,** 32–40.

[23] Weir, G. C., Knowlton, S. D., Atkins, R. F., McKennan, K. X., and Martin, D. B. (1976) Glucagon secretion from the perfused pancreas of streptozotocin-treated rats. *Diabetes* **25,** 275–282.

[24] Weir, G. C., and Bonner-Weir, S. (1990) Islets of Langerhans: the puzzle of intraislet interactions and their relevance to diabetes. *J. Clin. Invest.* **85,** 983–987.

[25] Kubota, N., Tobe, K., Terauchi, Y., Eto, K., Yamauchi, T., and Suzuki, R., et al. (2000) Disruption of insulin receptor substrate 2 causes type 2 diabetes because of liver insulin resistance and lack of compensatory beta-cell hyperplasia. *Diabetes* **49,** 1880–1889.

[26] Lingohr, M. K., Dickson, L. M., Wrede, C., Briaud, I., McCuaig, J.-F., Myers, M. G., and Rhodes, C.-J. (2003) Decreased IRS-2 expression in pancreatic $\beta$-cells (INS-1) promotes apoptosis, which can be compensated for by introduction of IRS-4 expression. *Mol. Cel. Endocrinol.* **209,** 17–31.

[27] Kulkarni, R. N., Winnay, J. N., Daniels, M., Bruning, J. C., Flier, S. N., and Hanahan, D., et al. (1999) Altered function of insulin receptor substrate-1-deficient mouse islets and cultured beta-cell lines. *J. Clin. Invest.* **104,** R69–75.

[28] Kulkarni, R. N., Roper, M. G., Dahlgren, G., Shih, D., Kauri, L. M., Peter, J. L., et al. (2004) Islet secretory defect in insulin receptor sustrate 1 null mice is linked with reduced calcium signaling and expression of sarco(endo)plasmic reticulum Ca2+-ATPase (SERCA)-2b and −3. *Diabetes* **53,** 1517–1525.

